# Chimpanzees (*Pan troglodytes*) Fail a What-Where-When Task but Find Rewards by Using a Location-Based Association Strategy

**DOI:** 10.1371/journal.pone.0016593

**Published:** 2011-02-16

**Authors:** Marusha Dekleva, Valérie Dufour, Han de Vries, Berry M. Spruijt, Elisabeth H. M. Sterck

**Affiliations:** 1 Behavioural Biology, Utrecht University, Utrecht, The Netherlands; 2 Institut Pluridisciplinaire Hubert Curien, Centre National de la Recherche Scientifique, University of Strasbourg, Strasbourg, France; 3 Ethology Research, Biomedical Primate Research Centre, Rijswijk, The Netherlands; Université Pierre et Marie Curie, France

## Abstract

Recollecting the what-where-when of an episode, or episodic-like memory, has been established in corvids and rodents. In humans, a linkage between remembering the past and imagining the future has been recognised. While chimpanzees can plan for the future, their episodic-like memory has hardly been investigated. We tested chimpanzees (*Pan troglodytes*) with an adapted food-caching paradigm. They observed the baiting of two locations amongst four and chose one after a given delay (15 min, 1 h or 5 h). We used two combinations of food types, a preferred and a less preferred food that disappeared at different rates. The subjects had to base their choices on the time elapsed since baiting, and on their memory of which food was where. They could recover either their preferred food or the one that remained present. All animals failed to obtain the preferred or present foods above chance levels. They were like-wise unsuccessful at choosing baited cups above chance levels. The subjects, thus, failed to use any feature of the baiting events to guide their choices. Nonetheless, their choices were not random, but the result of a developed location-based association strategy. Choices in the second half of the study correlated with the rewards obtained at each location in the first half of the study, independent from the choices made for each location in the first half of the study. This simple location-based strategy yielded a fair amount of food. The animals' failure to remember the what-where-when in the presented set-up may be due to the complexity of the task, rather than an inability to form episodic-like memories, as they even failed to remember what was where after 15 minutes.

## Introduction

The conscious re-experience of past events and the anticipation of future ones is ascribed to the ability of mental time travel into the past and future [1]. This advanced ability enables our own species the use of detailed knowledge from past personal experiences to meet current demands. Over the last ten years, innovative research has shown that, like humans, some animal species can remember certain features of experienced episodes as well as prepare for anticipated future events, albeit in a more limited manner [2]. Behavioural paradigms investigating episodic-like memory, a simplified version of the human episodic memory system, have led researchers to question the belief that animals are unaware of their past and future and might be “stuck in time”[3].

The episodic memory system forms together with the semantic system the declarative subdivision of the human long-term memory system [4], [5]. Both systems are characterised by the conscious retrieval of stored information. Whereas the semantic system stores facts and concepts acquired over several exposures, the episodic system consists of events formed after single exposure. Tulving [6] originally defined episodic memory as storing detailed information about the temporal and spatial features of a unique episode (the what-where-when), but later added, that such memories were episodic when accessed by conscious re-experiencing of the encoding event, or so called mental time travelling [7]. Investigating episodic memory in animals is constrained by their inability to verbally communicate their re-experience of an episode. Researchers have shown that animals remember important characteristics of experienced episodes, and, thus possess a system similar to the human episodic memory called episodic-like memory [2], [8]–[10] or what-where-when memory [11]. While these results fit Tulving's original definition of episodic memory [6], such behavioural paradigms cannot determine whether the animals experience conscious recollection [8], [12], [13].

Using a food-caching task, Clayton and Dickinson [13] tested western scrub-jay's (*Aphelocoma californica*) ability to form episodic-like memories. The animals cached two foods, preferred but perishable wax worms, and less favoured but not perishable peanuts. When recovering their caches, they tended to search for wax worms if only a short time had passed, but switched to the less-preferred peanuts if a long time had elapsed since caching. Thus they were successful at distinguishing the type of food they cached, its location and how long ago they made their caches [13]. Various studies have shown that other corvids: magpies (*Pica pica*) [14], black-capped chickadees (*Poecile atricapillus*) [15], as well as mammals: rats (*Rattus sp.*)[16]–[20], mice (*Mus musculus*)[21], and meadow voles (*Microtus pennsylvanicus*)[22] are able to recall the what-where-when of similar events. However, the jays' behaviour in the Clayton and Dickinson study [13] could be alternatively explained by a simple learned rule: search for the preferred wax worms if little time has passed, but avoid the worms if long time has passed since caching [23]. To exclude this option, a third food and time interval were added to the set-up. Jays now had to adjust their searches based on the what-where-when in six different conditions. They showed remarkable flexibility and appropriately adjusted their searches[23]. Clayton and colleagues have since reinforced and extended these results through further experimental investigations. They showed that the jays also can integrate the content of what-where-when episodes into a single memory; that they can flexibly update information about the decay rate of the foods [8], [23]; that they are sensitive to who observes them while caching [24]; and that they can even plan for future needs [25].

While considerable effort has been invested in the study of corvids' and rodents' episodic memory, few attempts have been made to test episodic-like memory in primates. This is surprising, as apes are known to possess advanced cognitive abilities [26] and show long term memory [27], [28]. Additionally, experimental and observational reports have shown that chimpanzees and other great apes are able to plan for the future [29]–[32]. Several studies with human subjects have established that planning and episodic memory share neural resources [1]. The few existing studies of primates episodic memory are burdened with potential alternative explanations of their findings and all have utilised different approaches [33]. This makes a comprehensive comparison of their findings difficult. The mnemonic ability of a single lexigram-proficient chimpanzee was examined by means of a free-recall paradigm [34]. Sixteen hours after an item was hidden in a large outside area, the chimpanzee obtained the attention of a naïve caretaker and, with the aid of the lexigram, led him to the items. The time interval used is impressive in length, and, informative about this animal's long term mnemonic ability. However, as the structure of the memory content is not tested [8], the results say little about the animals ability to form and recall episodic-like memories. An alternative approach to episodic memory in non-humans was examined by Schwartz and colleagues [35]. They tested a single gorilla (*Gorilla gorilla gorilla*) that indicated which food it had recently received from which caretaker by handing over the correct picture card [36]. The same gorilla was also tested with novel actions, persons and objects as well as with the temporal order of events. In all contexts, the gorilla returned the correct card above chance levels [35], [37]. These findings demonstrate knowledge of unique past events. Whether the animal truly recalled the details of the event or simply returned the card representing the most recent, and thus familiar, event remains open to discussion [35]. In monkeys, an adapted version of the Clayton and Dickinson [13]set-up examined the what-where-when components of memory with rhesus macaques (*Macaca mulatta*)[38]. The macaques were able to remember the locations of two foods for up to 25 hours, but failed to recognize that only the less preferred food was palatable after a long delay. More recently, three great ape species (*Pan paniscus, Pan troglodytes* and *Pongo pygmaeus*) were likewise tested on the what-where-when features of food hiding events by adapted paradigms of the Clayton lab [39]. In their first experiment the authors showed that the apes were able to remember where and when two types of foods were hidden by selectively choosing the perishable food item after a short interval, but switching to the non-perishable food following a long delay. However, the findings of this experiment could be explained by the same rule-based learning mentioned above [23]. To examine whether the three components (i.e. what, where and when) were structured into a single memory the authors further showed that the animals encoded two baiting events in an integrated fashion [39]. However, these findings can also be explained by the animals following the same rule, admittedly with impressive flexibility. To exclude rule learning, the experimental set-up could be complemented with an additional food and time interval, as was used successfully with corvids [23].

In the present study we adapted the extended three food and time interval paradigm of Clayton [23] to test memory of what-where-when in chimpanzees (*Pan troglodytes*). This paradigm is the only one that allows a comparison of behaviour between a control and a test group, while evaluating an animal's behaviour across multiple conditions and facilitating the exclusion of simple rule-based learning or familiarity processes. We determined the chimpanzees' preferences for three foods, trained them to retrieve a preferred food from four potential locations, and showed them that the three foods had different rates of disappearance over time. We tested whether the animals integrated this knowledge, which would allow them to retrieve their preferred food, or the food remaining present in six different food and time combinations. We predicted the animals would choose the preferred food on trials where both foods remained present, but switched to the less preferred food on trials where the preferred food had disappeared. If the animals adjusted their behaviour depending on the amount of time that passed since hiding, while remembering where each food type was hidden it would show they were able to remember the what-where- when of a given food hiding event.

## Materials and Methods

### Ethics

All training and testing was conducted as a part of the chimpanzee enrichment program of the Biomedical Primate Research Centre (Rijswijk, the Netherlands) and involved only positive reinforcement. The research activities were fully integrated into the daily routine and required no additional manipulation of individuals. The chimpanzees were not deprived of food and water at any stage. In addition, the individuals could choose not to participate in any individual trial, yet all individuals were rewarded with a small treat at the end of each trial, regardless of their level of participation in the task. The individuals showed a constant willingness to interact with the researcher, indicating that the tasks improved the well-being of the chimpanzees and that our efforts to minimize any potential suffering were successful. Therefore, the research offered positive stimulation for the individuals and follows the Weatherall Report recommendations for good welfare. The study was conducted in compliance with all relevant Dutch laws and in agreement with international and scientific standards and guidelines. Due to the non-invasive character of the study and absence of potential discomfort no additional permission from the institutes animal experiment committee was required. By definition following the Dutch Animal Experimentation Act, an animal experiment is undertaken with a scientific purpose and affects animal welfare. This study was not considered an animal experiment because animal welfare was not affected (enhanced if anything). This was so assessed by the Biomedical Primate Research Centre Animal welfare officer.

### Subjects

Nine individuals from three chimpanzee groups (named P, F and D) housed at the Biomedical Primate Research Centre (Rijswijk, the Netherlands) participated in this study (Table 1). The groups consisted of test individuals as well as several additional animals that were not tested due to inconsistent participation during the training stages. The participating animals had been living together for a minimum of 2 years and all three groups were socially stable. Because all training and testing involved only positive reinforcement, the individuals could choose not to participate in any individual trial. This is reflected in the different number of trials completed between the subjects.

**Table 1:** Study subjects age, group affiliation and rearing history.

Name	Age	Groups		Rearing history	
		Social	Test/Control	Hand reared	Mother reared (until age)
Claus	14	P	T	X	
Emanuel	17	P	T		X (2y)
Freek	14	P	T		X (1.5y)
Linda	22	F	T	X	
Marlis	26	F	T	X	
Paul	14	P	T		X (2y)
Rene	14	P	T	X	
Denis	23	D	C		X (7mo)
Regina	40	D	C		X (unknown, wild born)

P, F and D stand for names of the three different social groups the subjects belonged to. T denotes test group and C control group.

The three social groups were housed in similar enclosures with both inside and outside compartments. The outside compartment consisted of one large area (size approx. 87 m^3^). The inside compartment consisted of two large “play rooms” (for groups P & F approx. 75 m^3^, for group D app. 57 m^3^) as well as several individual cages (size approx. 1.6 m^3^) arranged in a row on one side of the play rooms. The groups had no visual access to each other's inside compartments. All chimpanzees had been trained to enter the individual cages as a part of their daily routine for feeding, cleaning and veterinary procedures. The outside compartments were connected onto a yard, arranged next to each other in a row allowing limited visual access between the social groups. Training and testing was performed in all compartments of the enclosures, depending on the stage of the study.

The animals were fed three times a day on a diet of chow, bread, fruit & vegetables. In the morning and afternoon the feeding took place in the individual cages, while at midday they were fed in the common parts of the enclosures. Water was available *ad libitum* throughout the study.

### Study design

The study began with a pre-training stage. We established that the animals preferred apple sauce and yoghurt over red bell peppers respectively. In the first of the two training stages we trained the animals to point to one of four distinctly marked locations in order to receive its content. For the second training stage, the subjects were divided into a test and a control group. Test animals were shown that the preferred apple sauce and yoghurt disappeared at different rates, while the less preferred red bell peppers always remained present. Control animals experienced that all three test foods always remained present. On each test trial the researcher showed the animal in which out of four differently coloured locations, two foods were hidden. After a delay of 15 min, 1 h or 5 h the animal was asked to point out which cup they wanted to receive. On each trial two foods were hidden in fixed combinations of either apple sauce and red bell peppers, or yoghurt and red bell peppers. The control group always experienced that both of the hidden foods were present at recovery, while the test group experienced that apple sauce disappeared after 1 hour and the yoghurt after 5 hours. Following the rationale of the Clayton [23] study we predicted that the animals in the control group would always choose the preferred food out of the two, while the test group would choose the preferred food on trials where both foods were present, but switch to less preferred food on trials where the preferred food had disappeared. Table 2 gives an overview of the predictions for the control and test group at 15 minutes, 1 hour and 5 hours. The choices of the control animals would demonstrate the animals ability to form long term memories for what is where, while the switch in choices of the test group would demonstrate their ability to integrate the what, where and when of the baiting episodes.

**Table 2:** Overview of the predicted choices for the test and control group.

Time interval	Apple sauce & Red bell peppers		Yoghurt & Red bell peppers	
	Control group	Test group	Control group	Test group
15 min	Apple sauce	Apple sauce	Yoghurt	Yoghurt
1 h	Apple sauce	Red bell peppers	Yoghurt	Yoghurt
5 h	Apple sauce	Red bell peppers	Yoghurt	Red bell peppers

### Pre-training procedure: Food preferences

The animals were tested on their preferences between three foods: apple sauce diluted with water 2:1, low fat natural yoghurt diluted with water 1:1 with three tablespoons of fruit syrup per litre and red bell peppers in 2×2 cm pieces. The foods were presented in two fixed combinations: apple sauce and red bell peppers or yoghurt and red bell peppers, which were pseudo-randomly varied between the daily sessions. This pre-training stage was conducted in the mornings following the animals' breakfast while they were already separated in individual cages. The foods were presented in familiar red paper cups (0.16l) containing one kind of food: one piece of red bell pepper, one spoonful of the yoghurt, or the apple sauce solution.

At the start of each session the animals received a taste of the two foods to establish their motivation to feed and to ensure they knew between which two foods they were choosing. Following consumption, the researcher presented two cups containing the foods. The two cups were tilted towards the animal and approximately 30–40 cm apart. The animals indicated their choice by reaching out for one cup. They received the chosen cup and could consume the food. In case an individual did not indicate its choice the researcher removed the cups and presented them again a few seconds later. If the animal remained unresponsive the session was terminated. Only data from sessions where the animals completed six trials were used for analysis.

On each day a combination of the same two foods was presented in six trials, with the side of presentation counterbalanced. Each food combination was tested on five to eight daily sessions, with all nine animals completing a minimum of 30 trials (mean = 36.7, SD = 3.5) per combination. The individuals did not complete the same number of trials. In order to demonstrate a significant preference of one food over the other, we set as a criterion that the animals should chose the preferred food in at least 70% of the trials. A binomial test with 21 positive choices out of 30 (70%) would show a significance of p = 0.046. Based on these choices we determined their preferences between apple sauce, yoghurt, and red bell peppers.

### Training procedure part one: Pointing

This part of the training was conducted while the animals were separated in their individual cages following breakfast. Two locations were baited, out of initially two and later four possible locations. The four locations were marked by cup holders of different colours and patterns that were located in the corners of an upright held square grid. At this stage we used foods other than those in the final test, i.e. bread or cookies as the preferred food and carrots as the less preferred food. These foods were selected based on the recommendations of the animals' caretakers.

Each session commenced after the animal consumed a small piece of both foods. The researcher first showed the animals that the cup holders were empty by tipping them upside down. Then, two holders were baited, each with a cup containing one food. The remaining two holders were treated with the same hand motions as the baited ones; however empty cups were inserted into them. During the baiting the researcher used verbal cues to encourage the animals to pay attention to the baiting. In case the animals looked away or were otherwise distracted, baiting at that location was repeated. The four holders were then covered with opaque lids. The metal grid was then held upright close enough for the animals to reach for, but not touch, one of the holders. The animals were, if necessary, verbally encouraged to make a choice and received the cup they indicated. If the animals did not point at any location the researcher stepped back and presented the holders again after a few seconds. If the animal remained unresponsive the trial was scored as no choice and was excluded from analysis. When individually separated, group members could see which cup holder their neighbours chose. This information could influence their choices. To prevent such visual cues the metal grid was either presented inside a large box (group P) or the researcher positioned herself at an angle so her back would function as a visual barrier (groups F and D).

Each training day consisted of one session with four trials per individual. All four locations were overall, baited approximately equally often in a pseudo-random order (mean number of baiting events per cup = 25, SD = 2.31). The animals were trained on ten to thirteen days with four cups, and all completed a minimum of 40 trials (mean = 50.00, SD = 4.28). For the animals to pass this training stage we set as a criterion that they should make at least 60% choices for the cup containing the preferred food. At 60% of correct choices, a Chi-square test with 40 trials (minimum completed) and expected choice of 25% would show a significance of p<0.001. The animals were then considered proficient at indicating the one location out of four that contained their preferred food, as well as at understanding the connection between the baiting and the choosing.

### Training procedure part two: Temporal properties of food

This part of the training was conducted in front of the entire social groups in the late morning. Depending on the cleaning routine, that restricted which parts of their enclosure the animals could access, the foods were either presented in front of the outside, or the inside enclosures. This ensured that as many individuals as possible witnessed the presentation of the foods.

The researcher first encouraged the individuals to come into the appropriate room by calling their names. All animals received a small amount of the test food to ensure they knew which food it was. A large amount of the test food placed in cups was left in front of the enclosures in plain view. The researcher left and returned after the predetermined time intervals of 15 min, 1 hour or 4 hours and gave the animals the cups. These either still contained the foods or were empty depending on the time interval and whether the animals belonged to the test or the control group.

For the members of the control group all three foods were always present upon recovery. For the members of the test group, however, the cups' content was manipulated so that the yoghurt and apple sauce were either present or absent at recovery. This manipulation was achieved by the following: prior to presentation the cups were either filled with the yoghurt or apple sauce solution, or left empty, but always covered with cling-film secured on top of each cup. The top of the cling film was covered with a layer of either apple sauce or yoghurt. This ensured that upon visual inspection the cups appeared full. These cups were presented to the animals and left in front of their enclosures. Once the designated time interval passed the researcher removed the cling film with her back to the animals' enclosure, so the subjects were unable to see her actions and then distributed the cups to the present animals. The animals would thereby experience that the foods disappeared immediately prior to them receiving the cups. The researcher would, for example, prepare full cups of apple sauce for the 15 minute interval and empty cups for the 1 and 4 hour intervals. The test group members were, thus, able to experience that, depending on the time interval, a given food could either be present or disappear, while the control animals experienced the same procedure but never experienced that the foods disappeared.

On each day one food was presented during one time interval and given to the animals after approximately 15 min (range 0:09–0:14), 1 hour (range 0:51–1:14) or 4 hours (range 3:46–4:09). The foods were first presented in a descending order of time intervals (4 hours, 1 hour and 15 minutes) and later ascending (15 minutes, 1 hour, 4 hours) order. First the red bell peppers were presented, second the yoghurt and thirdly the apple sauce. After this all three foods were presented again, each at 4 hours, 1 hour and 15 minutes.

### Testing procedure

The testing was performed while the animals were separated in their individual cages. Each session consisted of two parts; food hiding and food recovery. During both parts two persons were present; MD and a familiar animal caretaker. One person was hiding or recovering the food, the other was videotaping the trial. The two persons always switched roles within a session, to ensure that the person performing the recovery was unaware of the actual location of the foods and thus unable to cue the animals.

The hiding and recovery procedures were essentially the same as the procedure described above for pointing training. Metal grids with four new distinctly coloured holders were used. Once the foods were hidden in front of each animal, the grid was placed in front of that individual's cage. The foods were hidden for approximately 15 min (range 0:08–0:21), 1 h (range 0:56–1:24) or 5 h (range 4:58–5:38). Due to logistical reasons the long time interval was somewhat longer than the one under the training of temporal properties. We reasoned that if the animals successfully learned the foods temporal properties during training, then extending this interval should not influence the direction of the animals' responses.

The animals remained in the individual cages for the 15 minute and 1 hour intervals, but were released and re-entered the individual cages on the 5 hour intervals. We could not ensure that they entered the same individual cage at recovery as they occupied during the hiding. Therefore, we gave each animal's grid an individual colour code, which was a large coloured paper placed in the middle of their metal grid. Before each hiding, the subjects were given a small paper of the same colour with honey or peanut butter to attract their attention to the colour. During the 5 hour intervals the animal's grids remained in front of the cages in which the animals were during the food hiding. Once the animals re-entered the cages for the food recovery the grids were moved so that each animal was situated in front of its own grid. During the 15 minute and 1 hour intervals the girds remained in front of the same individual cages, as the animals were not released.

**Figure 1:**
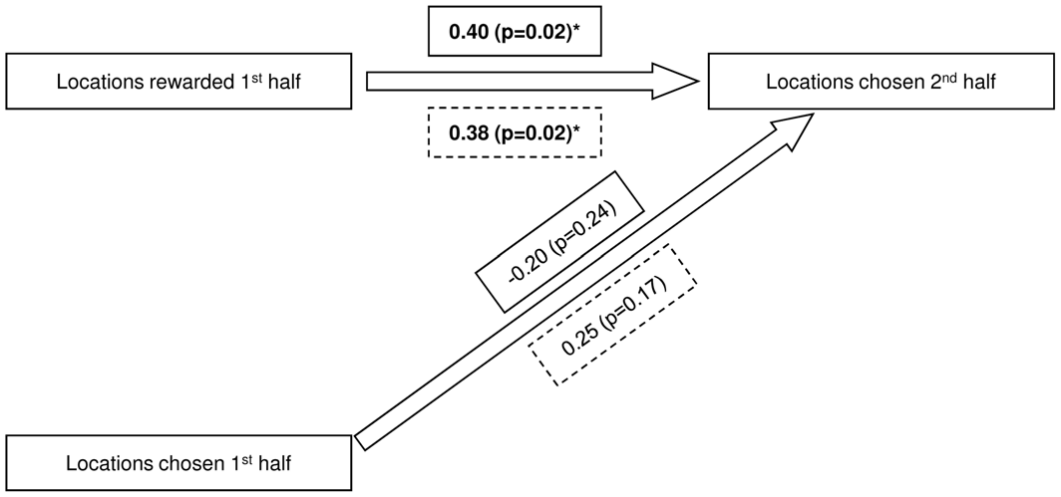
Manipulating the temporal properties of the foods during testing. Panel A shows on the left the four different cup holders (a, b, c & d) attached to the metal grid. On the right are the eight identical plain cups that were inserted into holders a–d in front of each subject. Two stacked cups were inserted into each cup holder. Only two of them contained food. In this example, a 1 hour apple sauce and red bell peppers trial, cup two contained red bell pepper and cup eight contained apple sauce. The remaining 6 cups were empty. Panel B shows that four cups were removed from the holders with the researcher's back to the subject, immediately after the food hiding. One of the plain cups was removed from each holder. In this example, upon recovery, holder a still contained the red bell pepper, while the apple sauce from holder d had disappeared.

For the members of the test group the yoghurt and apple sauce disappeared from the baited locations after the 1 and 5 hour intervals. This was achieved in a different way than during training (see also Figure 1). Four coloured holders were permanently attached to the same locations on the grid. Two stacked identical plain cups were inserted into each of these holders during food hiding. Two holders were baited with two stacked cups each, where one of the cups contained the test food, while the other was empty. The remaining two holders were baited with two empty stacked cups each. The animals observed the hiding of the foods in the stacked cups, into the holders, and the application of opaque lids. However, as the cups were stacked one inside the other, the animals just saw the baiting of the four holders with two empty cups and two cups containing food. After the hiding, the researcher placed the grid in front of each individual's cage and turned her back to the animals. She then (always in the same sequence) removed one of the stacked cups from each of the holders. Depending on the time interval she either removed the cups containing the food, or the empty cups. For example in a 15 minute interval four empty cups were removed, and thus both foods were still present at recovery. However at a 1 hour interval (Figure 1) the cup containing the apple sauce was removed and upon recovery only the red bell peppers remained.

After the retention interval the animals were presented with their metal grids and verbally encouraged to reach for one location. They then received the inserted plain cup and its content. If the animals would not indicate a choice the grid was removed and presented again after a few seconds. If they remained unresponsive the trial was scored as no choice trial and excluded from the analysis.

At the beginning to the testing period we first familiarized the animals with the testing procedure. Each animal received one hiding and recovery session at each of the three time intervals. During this familiarization we used the same foods as during pointing training.

The 15 minute and 1 hour intervals took place, either in the morning or in the afternoon, following the morning or evening meal. The hiding in the 5 hour intervals was performed in the morning and the foods were recovered in the afternoon, both following the animal's feeding time. The animals received a maximum of three testing sessions each day. Each animal received between seven and twelve trials of both food combinations in each of the three time intervals (mean = 9.52, SD = 1.30). Four test trials (from four different individuals) were excluded from the analysis due to researcher error during testing, where incorrect foods were present during recovery. For each of the possible six food and time combinations the position of the foods was counterbalanced. All four locations were baited approximately equally often with both foods for each animal (mean baiting events per cup = 28.5 SD = 2.53). The sequence in which the food and time interval were presented was pseudo-randomized.

### Analysis

During training and testing we manually scored the choices the animals made and whether they consumed the obtained food. During the training of the foods temporal properties we scored the group members present, both, when the foods were presented and when they were given. All test trials were videotaped as well as recorded manually after each trial. Data were entered into Excel from the observation sheets and, in case of inconsistencies, confirmed from videotapes. The data were analyzed with SPSS 16 and MatMan 1.1 (Noldus Information Technology, Wageningen) [40]. Each animal's behaviour was tested individually, reasoning that just one animal's success would be of importance. We used Chi square tests and row-wise matrix correlation tests[41]. All statistical tests were two-sided at a critical alpha of 0.05. We used the standard Bonferroni correction for multiple comparisons.

## Results

### Pre-training: Food Preferences

We scored the animal's choices in the two different food combinations: apple sauce and red bell peppers, and yoghurt and red bell peppers. All individuals chose apple sauce over red bell peppers in more than 70% of the trials (mean = 96.60%, SD = 4.77). All individuals except one (Marlis) chose yoghurt over red bell peppers in more than 70% of the trials (mean  = 87.73%, SD = 18.89; excluding Marlis: mean  = 93.14%, SD  = 10.33). Marlis was hereafter excluded from the analysis. None of the animals showed a significant preference for a particular side in either food combination (Binomial test, all individuals p>0.31). The animals were thus not guided by a side bias in their choices. All remaining individuals (i.e. except Marlis) showed a significant preference for apple sauce over red bell peppers and for yoghurt over red bell peppers.

### Training part one: Pointing

All individuals chose the locations containing the preferred food in more than 60% of the trials (mean = 81.55, SD = 9.68). The less preferred food was chosen on average in 5.34% of the trials (SD = 4.27) while the two empty cups were chosen on average in 13.06% of the trials (SD = 7.99). This demonstrates that all eight individuals were able to clearly discriminate and indicate the location of the preferred food.

Additionally, to assess the animal's motivation for choosing a particular cup, we scored whether they consumed the obtained foods. When obtained, the preferred food was consumed on 100% of the trials, while the less preferred food was consumed in 14.3% of the trials. This further indicated that the animals distinguished between the qualities of the different rewards and wanted to obtain the preferred food.

### Training part two: Temporal properties of food

We determined the percentage of trials where the individuals were present, both when a particular food was placed outside their cage, and when it was handed out (mean  = 92.28%, SD = 13.39). Furthermore, we looked at each individuals presence on the informative trials, defined as those where any of the foods had disappeared. The animals received a total of 5 informative trials in each food and time combination. All individuals were present on minimum 80% of the informative trials. For apple sauce that was at 1 hour: mean  = 97.50, SD = 7.07 and 4 hours: mean  = 97.50, SD = 7.07. For yoghurt that was at the 4 hour interval: mean  = 97.50, SD = 7.07. We assumed that the animals were given sufficient opportunity to learn the temporal properties of the presented foods.

### Testing: what-where-when choices

We hypothesized that the animals' behaviour in the test could be guided by three different choice strategies, which we consider in turn. The first strategy we investigated was whether the animal's choices were guided by the principles of the paradigm; so they in each of the six time and food conditions successfully integrated which food was hidden where as well as the time passed since hiding (Table 3). We examined whether the animals made more of such what-where-when choices than expected by chance (25%). We performed the analysis on three different levels: each animal's choices in all of the conditions, at each of the three time intervals irrespective of the food combination and their choices in each time and food condition separately.

**Table 3:** The number of trials and the what-where-when and what-where choices per animal in each time and food combination.

Food and time combination		Test group						Control group	
		Claus	Emanuel	Freek	Linda	Paul	Rene	Denis	Regina
Apple sauce & Red Bell Peppers (15 min)	# Trials	11	9	11	7	11	11	11	12
	WWW choices	2 (18)	1 (11)	6 (55 1)	3 (43)	4 (36)	3 (27)	3 (27)	2 (17)
	WW choices	4 (36)	4 (44)	8 (73)	5 (71)	5 (45)	5 (45)	6 (55)	5 (42)
Yoghurt & Red Bell Peppers (15 min)	# Trials	11	11	11	7	11	11	10	11
	WWW choices	3 (27)	2 (18)	1 (9)	1 (14)	4 (36)	3 (27)	3 (30)	4 (36)
	WW choices	6 (55)	4 (36)	7 (64)	3 (43)	7 (64)	4 (36)	5 (50)	7 (64)
Apple sauce & Red Bell Peppers (1 h)	# Trials	8	8	8	11	8	7	9	9
	WWW choices	3 (38)	0 (0)	2 (25)	6 (55 2)	2 (25)	2 (29)	1 (11)	3 (33)
	WW choices	7 (88)	3 (38)	5 (63)	8 (73)	2 (25)	2 (29)	6 (67)	5 (56)
Yoghurt & Red Bell Peppers (1 h)	# Trials	10	10	10	8	10	10	10	10
	WWW choices	1 (10)	5 (50)	3 (30)	3 (38)	3 (30)	3 (30)	3 (30)	5 (50)
	WW choices	4 (40)	5 (50)	6 (60)	6 (75)	5 (50)	5 (50)	6 (60	7 (70)
Apple sauce & Red Bell Peppers (5 h)	# Trials	9	9	9	8	9	8	8	8
	WWW choices	5 (56 3)	2 (22)	4 (44)	2 (25)	3(33)	3 (38)	1 (13)	1 (13)
	WW choices	7(78)	4 (44)	5 (56)	5 (63)	5 (56)	4 (50)	3 (38)	2 (25)
Yoghurt & Red Bell Peppers (5 h)	# Trials	10	10	10	8	10	9	9	10
	WWW choices	3 (30)	3 (30)	3 (30)	2 (25)	3 (30)	1 (11)	3 (33)	4 (40)
	WW choices	6 (60)	5 (50)	7 (70)	4 (50)	5 (50)	4 (44)	4 (44)	6 (60)

1 exact Chi-square test: χ^2^ = 5.12, df = 1, p = 0.034.

2 exact Chi-square test: χ^2^ = 5.12, df = 1, p = 0.034.

3 exact Chi-square test: χ^2^ = 4.48, df = 1, p = 0.049.

WWW stands for what-where-when choices and WW for what-where choices. WWW choices resulted in obtaining either the present or preferred food (according to the paradigm's predictions for each combination). WW choices were those made for either of the two baited cups, regardless of whether the food was still present at recovery. The animals had a 25% chance of making the correct WWW choice, for the WW choices this chance was 50%. Percentages are given in brackets. Significant values before Bonferroni correction are indicated by footnotes. None of the values remained significant after the Bonferroni correction.

When all the food and time conditions were pooled together, none of the animals made significantly more what-where-when choices than expected by chance (exact Chi-square test, df = 1, all individuals p>0.23). Next, to investigate whether the length of the retention interval influenced the animal's success rate; we pooled the number of what-where-when choices made at each of the three time intervals irrespective of the food combinations. One of the animals (Linda) made in the 1 h condition significantly more what-where-when choices (exact Chi-square test, χ^2^ = 5.07, df = 1, p = 0.03). However, after a Bonferroni correction for multiple comparisons this value was no longer significant. All the other animals were unsuccessful at all three time intervals (exact Chi-square test, df = 1, all p>0.11). Lastly, we looked at the what-where-when choices made in each time and food condition separately. Three different animals (Freek at 15 min, Linda at 1 h and Claus at 5 h) made significantly more what-where-when choices, all in the apple sauce and red bell pepper condition (statistics in Table 3). However, after a Bonferroni correction, none of the values remained significant. All other animals were unsuccessful (exact Chi-square test, df = 1, all p>0.13) in all food and time combinations. Thus, none of the animals made significantly more choices for their present or preferred food in any of the food and time combinations and, thus, all chimpanzees failed to pass the criteria of the food-caching paradigm.

### Testing: what-where choices

The second potential strategy we considered, involved determining whether the animals were successful at making what-where choices, considering either food type rewarding (Table 3). Success at these choices would indicate that the animals were in each trial remembering either of the two baited locations, but disregarding the temporal properties of the foods. As two cups were baited in each trial the animals chance success rate of making what-where choices was 50%, however, in trials where only one food remained present these choices would not result in a reward.

We investigated whether the animals were choosing the two baited cups more often than expected by chance (50%) when pooled together for all food and time intervals. One of the animals (Freek) made significantly more what-where choices (exact Chi-square: χ^2^ = 4.898, df = 1, p =  0.036; all other individuals exact Chi-square test, df = 1, all p>0.08). However, this value did not remain significant following a Bonferroni correction. We also tested the number of what-where choices the animals made at each of the three time intervals regardless of the food combinations. None were successful above the chance level (exact Chi-square test, df = 1, all p>0.06). All animals also failed to make significantly more what-where choices in each food and time combination separately (exact Chi-square test, df = 1, all p>0.07). The animal's choices were, thus, not guided by the distinction of which cups were baited and which were left empty in each trial.

As a measure of the animals' interest in the different food types we scored, whether or not they consumed the obtained foods. The preferred foods (apple sauce and yoghurt) were consumed in 98.5% of the obtained trials and red bell peppers were consumed in 97.2% of the obtained trials. Thus, during the testing stage the animals consumed any food they obtained regardless of preference, indicating they, in contrast to their behaviour during training, no longer discriminated between the qualities of the rewards.

### Testing: Location-based choices

The third possible strategy we examined was, that the animals' behaviour was directed by the formation of an association between a location and its potential to yield a reward. We called these the location-based choices. We considered both foods as a reward. Such choices would not be based on any information recalled from each baiting event, but simply on the different reward qualities of the four distinct locations.

First, we tested whether each individual showed a preference for a specific location, regardless of its content (Table 4), considering their choices from the entire testing period. All but one animal (Regina) showed a clear location preference (Table 4), as the number of choices they made for each location was significantly unequal, also after the Bonferroni correction. We also checked whether the number of rewards the animals obtained at each location differed (Table 4). All but one animal (Regina) were unevenly rewarded at each location (Table 4). After a Bonferroni correction, this remained significant for three individuals (Emanuel, Claus and Paul). As the animals were choosing certain locations more often than others, they also obtained more rewards from these locations.

**Table 4:** The number of times each animal chose and was rewarded at each location, separated for the first and second half of the study.

Name	Choice	Location 1		Location 2		Location 3		Location 4		exact Chi-square testdf = 3
		1st half	2nd half	1st half	2nd half	1st half	2nd half	1st half	2nd half	
Claus	Chosen	4	0	7	0	5	0	14	29	χ2 = 72.46, p = 0.001*
	Rewarded	0	0	4	0	2	0	7	12	χ2 = 35.96, p = 0.001*
Emanuel	Chosen	13	19	14	1	1	0	1	8	χ2 = 36.40, p = 0.001*
	Rewarded	4	7	3	0	0	0	0	4	χ2 = 14.45, p = 0.002*
Freek	Chosen	13	7	14	10	0	0	3	12	χ2 = 22.42, p = 0.001*
	Rewarded	6	5	7	3	0	0	2	7	χ2 = 10.27, p = 0.017
Linda	Chosen	5	4	11	17	7	1	2	2	χ2 = 28.14, p = 0.001*
	Rewarded	4	3	5	8	3	0	1	0	χ2 = 14.0, p = 0.003
Paul	Chosen	23	19	3	0	3	1	1	9	χ2 = 69.07, p = 0.001*
	Rewarded	10	6	1	0	2	1	0	5	χ2 = 21.56, p = 0.001*
Rene	Chosen	5	1	11	24	8	2	4	1	χ2 = 43.0, p = 0.0000*
	Rewarded	2	0	5	7	3	1	2	0	χ2 = 13.60, p = 0.003
Denis	Chosen	5	4	17	12	4	7	3	5	χ2 = 20.68, p = 0.001*
	Rewarded	2	2	10	5	3	4	1	3	χ2 = 10.80, p = 0.013
Regina	Chosen	8	6	19	6	3	7	0	11	χ2 = 9.47, p = 0.023
	Rewarded	4	4	12	2	2	2	0	6	χ2 = 7.0, p = 0.068

Significant values after Bonferroni corrections are indicated by*.

We were interested in whether this relationship between chosen and rewarded location could be a result of certain decision rules. We considered two possibilities: the win-stay lose-shift strategy and an associative learning process across the first half of the testing sequence.

We tested whether the animals based their choices on a win-stay, lose-shift strategy across the entire testing period, regardless of the time and food combinations. We counted for each animal how many times they performed the following behaviours: win-stay (if the chosen cup was rewarded irrespective of food type, the following choice is for the same cup), win-shift (if the chosen cup was rewarded, the following choice is for a different cup), lose-stay and lose-shift. By means of a Chi-square test for a 2×2 cross table we tested whether the chimps behaved consistently according to this win-stay, lose-shift strategy. For one of the animals (Emanuel) we did find a significant relationship (exact Persons Chi-square test: χ^2^ =  5.34, df = 1, p = 0.039), however, after a Bonferroni correction this value did not remain significant. No significant relationship was found for any of the other animals (exact Chi-square test, df = 1, all individuals p>0.16).

We then proceeded to investigate whether the number of times each location was chosen in the second half of the testing sequence, was influenced by which locations yielded food in the first half of the training sequence. To this end we counted for each chimpanzee the number of times each location was chosen in the first and second half of the testing sequence, as well as the number of times each location was rewarded in the first and second half of the testing sequence (Table 4).

**Figure 2:**
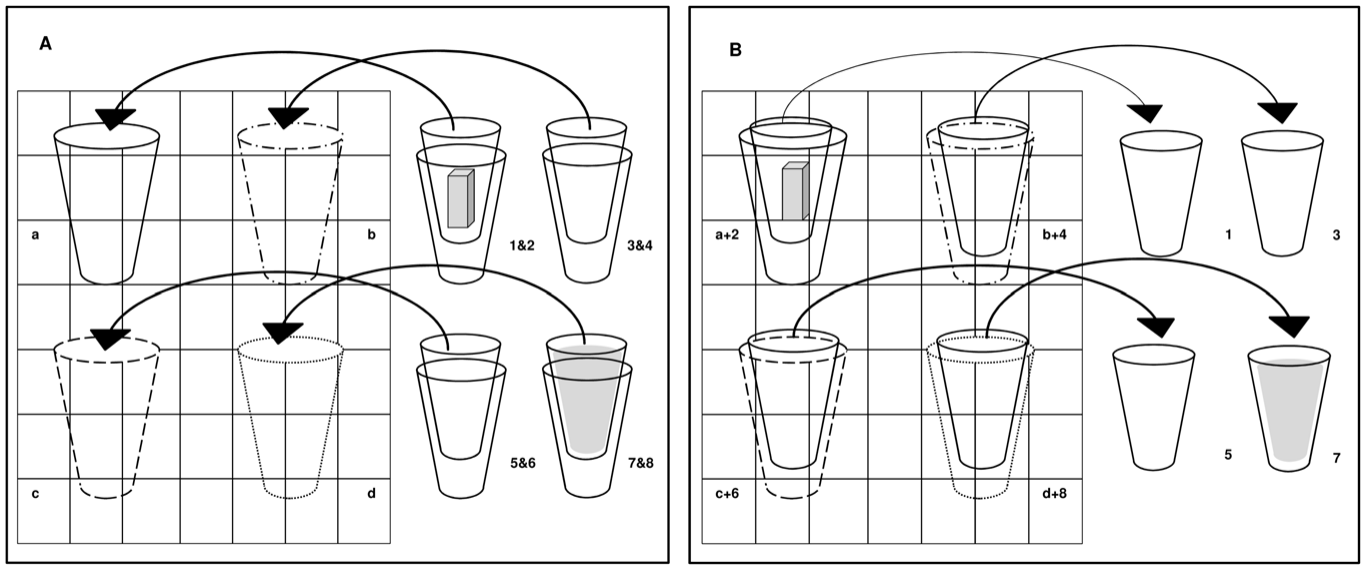
The relationship between chosen and rewarded locations. The locations chosen in the second half of the testing sequence depend on the locations rewarded in the first half of testing, not on the locations chosen in the first half of the sequence. Values in the closed line boxes are Kendall's tau_rw_ correlations; values in the dashed line boxes are partial Kendall's tau_rw_ correlations.* indicates significant values.

We used a row-wise matrix correlation [41]to test whether the number of choices for each location in the second half was related to the number of choices made for each location in the first half of the testing sequence. We obtained a positive non-significant Kendall's tau_rw_ correlation of 0.25 (p = 0.17). We then computed a partial row-wise correlation between the choices in the first and second half, controlled for the number of rewards obtained in the first half, and found that the previous positive correlation completely disappeared (and even became negative): partial Kendall's tau_rw_ controlled for rewards obtained in the first half  = −0.20 (p = 0.24). Thus, the cup locations' choices in the second half were made independently from the choices made in the first half of the testing sequence (Figure 2).

We then investigated whether the number of choices made in the second half was related to the number of rewarded choices obtained in the first half. We found a significant positive Kendall's tau_rw_ correlation of 0.40 (p = 0.023). Next, we computed a partial row-wise matrix correlation to see whether this correlation remained when we controlled for the number of choices made in the first half. Indeed, the correlation remained virtually the same: partial Kendall's tau_rw_  = 0.38 (p = 0.024). This shows that, it was indeed the rewards obtained in the first half of the testing sequences, and not the location choices themselves, which influenced the number of location choices in the second half of the testing sequence (Figure 2).

### Location based choices in training

We revisited the pointing training trials to see whether a location-based choice pattern was already visible then. We tested whether each animal chose all four locations equally often, based on the total choices made for each location. Exact Chi-square tests showed that two of the animals (Rene and Emanuel) were not choosing all of the locations equally often; only one of which (Rene) remained significant after a Bonferroni correction (Emanuel: χ2 = 8.15, p = 0.044; Rene: χ2 = 17.69, p = 0.001). The remaining six animals did not preferentially choose one of the four locations (exact Chi-square test, df = 3, p>0.18).

We also tested whether each chosen cup was rewarded equally often for each animal. Exact Chi-square tests showed that only for one animal (Rene) the four locations were not rewarded equally often; however, this value did not remain significant after a Bonferroni correction (Rene: χ2 = 9.00, p = 0.030). The remaining seven animals were rewarded equally often at each location (exact Chi-square test, df = 3, p>0.44). Thus, just one out of eight animals showed a location based preference during training and none of the animals received significantly more rewards at any location.

We used a row-wise matrix correlation to investigate whether the location choices made in the second half of the training were correlated to the location choices in the first half of the testing. The correlation resulted in a Kendall's tau_rw_  = −0.089 (p = 0.652), demonstrating that the animals were not choosing the same locations under testing as under training.

## Discussion

The objective of this study was to investigate chimpanzees' episodic-like memory by means of a what-where-when food-caching paradigm[23]. All individuals failed to pass the success criteria for demonstrating episodic-like memory in our set-up. The chimpanzees, however, did develop a location-based association strategy, based on the experienced reward quality of the four locations. Through association and not episodic memory they were able to locate, not where rewards were hidden, but at which location they had a higher probability of finding them. This behaviour reveals an interesting strategy of how the animals, when exposed to a multitude of changing features through-out the testing sequence (what, where and when), focused on the stable locations of the cup holders and, by means of a simple strategy, obtained apparently sufficient rewards.

All eight individuals successfully demonstrated a clear order of preference between the test foods, were able to reliably point to the cup they wanted to receive, and witnessed the different foods' rate of disappearance. In the testing phase three of the animals appeared to make more what-where-when choices than expected by chance, but each individual at a different time interval. None of these values remained significant after the Bonferroni correction. All significant what-where-when choices occurred in the apple sauce and red bell pepper condition. This may be attributed to a stronger difference in preference between these foods (compared to the difference between yoghurt and red bell peppers), which may have motivated them to pay more attention to these foods' locations. Nonetheless, to fulfil the success criteria for episodic-like memory the same animal would need to exceed the chance level of what-where-when choices in at least two time intervals. Success at least two time intervals would demonstrate a switch in choice strategy based on the presence of the food types. The fact that the animals failed to make more what-where-when choices than expected by chance, means they failed to integrate the unique trial locations of both foods (what is where), together with the time passed since caching (when), and adjust their choices accordingly for either the preferred food types (after the short interval) or the present food types (after the long interval). This choice strategy poses the highest cognitive requirements to the animals. Importantly, it is precisely the complexity of the task that is essential to conclusively demonstrate the presence of this advanced cognitive capacity [23]. Previous work showed that great apes can solve a less complex paradigm involving what-where-when choices [39]. However, the demand on the flexibility and adjustment of behaviour is higher in our settings than in the previous study. Additionally, successful performance in this previous study may be ascribed to rule learning [23]. From the current literature, including present work, none of the tested great apes or other primate species matched the response of corvids as tested by Clayton and colleagues [12].

Several explanations could account for our chimpanzees' failure. Firstly, the animals may have failed to obtain the knowledge about the temporal disappearance of the test foods, or failed to integrate this with their what-where knowledge. Although of potential influence, we do not believe this to be the main explanation of our results. All of the animals showed a poor performance even at the two 15 minutes conditions in which none of the foods disappeared. Also, the two control animals (Denis and Regina) never experienced the foods temporal disappearance and still failed to make what-where choices above chance level. Two other potential explanations for the animals' failure are that they either do not possess the necessary cognitive ability, or that the executive demand imposed by our set-up was too high. In order to distinguish between these two alternatives, we first determined whether the animals' choices were based on any of the information given to them during the food hiding in each trial. This will illuminate which information provided by the set-up the animals were able to utilize.

We examined whether the animals were basing their choices on the ‘what was hidden where’ information, by looking at the so-called what-where choices. One animal did appear to make more choices for the two baited cups when all six conditions were considered together, however, the value did not remain significant after the Bonferroni correction. None of the other animals were successful above chance levels. This indicates that the animals were not basing their choices on the what-where information in each trial. Such poor performance contradicts other studies of chimpanzee long-term memory, in which chimpanzees were shown to remember the location of at least one food even up to 3 days [34], [39]. Our animals were also out-performed by rhesus macaques, who were able to remember what is where for up to 25 hours [38]. Again, none of the eight animals in our study performed above random chance even on remembering what is where for 15 minutes, a time interval that should not have exceeded the species' mnemonic capacity [27], [28], [34]. The animals were thus not utilizing the information provided during the food hiding as a cue for their searches during recovery. We parsimoniously suggest that the testing procedure placed too high a demand on our subjects. This could be a result of several factors. Our procedure assumes that the animals, at the least, understand that they can recover foods from the locations where they observed foods being hidden. In fact, during pointing training when the food hiding was immediately followed by the recovery, the animals were successful at indicating the location of their preferred food. However, at this stage the animals only had to remember the location of one food (the preferred one) and this information only needed to be stored in their working memory, as retrieval was immediate. In testing, when food hiding and recovery were separated by intervals, the animals' success level dropped. Possibly, the combination of the time intervals and the need to distinguish the location of two foods between four options, may have limited the quality of the encoding of where the foods were hidden. Due to logistical reasons the animals only received one habituation trial per time interval of the testing procedure. Increasing the amount of such habituation trials could facilitate better understanding that the hiding locations were reliable cues for the locations of the foods during recovery. Finally, in contrast to other primates studies [34], [35], [38], [39] our animals had previously only participated in one behavioural study and were therefore naive to the concept of “working” for food. This suggests that the testing of such complex abilities may require a large amount of training and experience with similar testing procedures. However, it is essential that the amount of training is appropriately balanced so that the animals test performance reveals their intrinsic capacities and not a trained response. The tested animals thus failed to use the information provided under the food hiding procedure to guide their behaviour when making their choices. They show no evidence of integration of the what-where-when elements and consequently do not show episodic-like memory in our study. Whether or not chimpanzees are indeed able to form episodic-like memories in the domain of food will need to be established in future studies. Future testing should ensure that the animals attend to the hiding procedure and that the necessary prerequisites for memory formation are present.

We further examined the pattern of the animals' choices, to determine whether it was different from random choice. We considered a potential choice strategy based on the static locations of the four holders. We found that seven out of eight animals developed a significant location-based preference. For three of these individuals this also coincided with a higher rate of rewards at these locations, despite the fact that all four locations were baited approximately equally often. The animals' initial preference could have been influenced by the cups colour or position relative to the subjects' eye level or hand used for pointing. This location preference was further self reinforced, as persistent choices for a given location resulted in relatively more rewards obtained there. Interestingly, these location-based preferences developed during the testing phase. We found that a win-stay lose-shift strategy did not reliably explain the development of these preferences. We considered a more general association-based strategy. When looking at the number of each animal's location choices in the first and second half of the testing sequence, we found no significant correlation between the number of times the chimpanzees chose each of the four locations in the first and second half of the study. In fact, when we controlled for the influence of the rewards obtained at each location in the first half the result was even a negative, non-significant correlation. This means that the animals were not choosing the same locations in the first and second half of the study, indicating a certain shift in the choices the animals made in the second half of the testing sequence, compared to the first half. Indeed, we found that the rewards the animals obtained in the first half influenced the choices made in the second half, even when we corrected for the choices made in the first half of the study. Consequently, choices in the second half depended on the number of rewards obtained at these locations in the first half of the test phase, but were independent from the number of cup location choices made in the first half. Thus, the animals' behaviour is best explained by a location-based associative learning strategy. The animals formed associations about the potential of the different locations to yield rewards. This knowledge about the reward values of each location was formed through several experiences in the first half of the testing sequence, and then used in the second half of the testing sequence to guide their choices. On average this strategy yielded, per individual, rewards in 45% of the trials received. Considering that the animals were tested with several trials per day, they obtained about one reward per day. Additionally, we noticed that the animals readily consumed any food they obtained during testing, indicating that, in contrast to their behaviour during training, they disregarded their food preferences. Since the chimpanzees were obtaining fewer rewards during the testing phase, the value of any food may have increased compared to the training trials. Given that this simple strategy resulted in a fair amount of obtained rewards, it is likely that the more difficult strategy in which the what, where and when had to be remembered, was not called upon by the animals.

In conclusion, we aimed to examine the chimpanzees' episodic-like memory by means of a what-where-when food-caching paradigm. Altogether, our chimpanzees showed a much poorer performance compared to scrub jays on a similar task [23] or compared to rodents, monkeys and apes on a simplified version of the task [17], [38], [39]. Nonetheless, we maintain that none of the to-date present work, excluding the one on scrub jays, validates the demanded criteria for demonstrating episodic-like memory in primates. In other great ape studies [34], [35], [39] the animals response can be explained by more parsimonious explanations than the capacity to flexibly integrate the what, where and when elements. While chimpanzees are known to possess most of the cognitive tools required to a-priori solve episodic-like memory tasks, evidence remains slim and our results stress that whenever simpler alternative strategies can be satisfactorily used, chimpanzees may well rely on these. Given their natural skills in food-caching and recovery, corvids, such as scrub jays may have a head start to successfully and flexibly solve this type of task. Further research with carefully designed set-ups will be required to detect the potential for similar skills in non-corvid species.
